# Divergent mechanisms of perceptual reversals in spinning and wobbling structure-from-motion stimuli

**DOI:** 10.1371/journal.pone.0297963

**Published:** 2024-02-21

**Authors:** Leo Poom

**Affiliations:** Division of Perception and Cognition, Department of Psychology, Uppsala University, Uppsala, Sweden; Technical University of Madrid, SPAIN

## Abstract

This study explores the visual phenomenon of random dot structure-from-motion (SFM), where the brain perceives 3D shapes from the coordinated 2D motion of dots. Observing SFM may lead to ambiguous depth relations that reverse back and forth during prolonged viewing. I demonstrate that different processes are involved in triggering perceived reversals for identical SFM shapes involved in spinning and wobbling motion. Durations of stable percepts were measured while human participants viewed the two SFM stimuli, and also a static Necker figure, and a wobbling Necker figure for two sets of 2.5 minutes each. The results showed that wobbling SFM resulted in much longer stable durations compared to the other stimuli. The durations for the wobbling SFM stimuli was not correlated with the spinning SFM, or the two Necker stimuli. Yet, such correlations were obtained between the other stimuli. It is known that reversals obtained while viewing spinning SFM stimuli involves bottom-up driven adaptation and recovery cycles between neural populations. This result suggests that wobbling SFM efficiently deactivates this process and targets other contributions to the reversals, such as top-down processes. In addition, biases observed in the first set disappeared in the second set implying influences of learning between the sets. Imagery vividness, which measures intrinsic top-down processes, was also scored but no correlation between scores in visual imagery and reversal rates were obtained. This research provides insight into the complex interplay between bottom-up driven adaptation-recovery cycles, and top-down processes in ambiguous perception.

## Introduction

Ambiguous visual stimuli are efficiently used to probe endogenous processes in the brain since the sensory experiences switch spontaneously back and forth over time although the same stimulus is presented. The exploration of ambiguous stimuli has captivated researchers, prompting investigations into the underlying neural processes that contribute to perceptual reversals. Furthermore, the utilization of ambiguous stimuli has provided a valuable avenue for examining the interplay between perception and personality, as well as clinical diagnoses, by correlating these factors with observed rates of perceptual reversals during observation. An example of such stimulus is random dot structure-from-motion (SFM) from which we perceive 3D shapes based on the coherent 2D motion of randomly arranged dots. Such stimuli are ambiguous in that the perceived depth relations switches back and forth during prolonged viewing. The classical stimulus, dominating in research on SFM, consist of dots randomly spread over a simulated spinning cylinder parallel projected on a flat 2D plane [1]. When observing the moving dots on the 2D plane the 3D structure of the cylinder is recovered, but it is perceptually ambiguous. The perceived spinning direction (or the sign of depth) reverses spontaneously back and forth during prolonged observation. In a previous study I showed that the reversal rate of SFM stimuli slow down markedly when a wobbling motion (oscillating back and forth), rather than constant spinning in the same direction is used, possibly due to lowered rates of adaptation when spinning direction and 3D orientation of the object changes during observation [2]. We recently proposed that the reversals in perceived sign of depth when observing such wobbling SFM is mainly driven by endogenous top-down driven processes, i.e. the mind alternates between hypotheses about which distal 3D structure causes the motion in the 2D image [3]. We found support for this hypothesis in that an endogenously generated imagined prime strongly influenced the perceived depth order of the wobbling SFM. Still, the scores from imagery questionnaires, estimating the vividness of endogenously generated visual form, could not predict this influence. It is not known whether reversals of the wobbling SFM involve the same process of adaptation and recovery cycles as for the spinning SFM. It may be that this process is just slowed down when wobbling motion is used. Alternatively, a slower top-down driven process may dominate for the wobbling SFM.

Here, I further investigate the hypothesis that a wobbling motion of an SFM stimulus selectively target a slower top-down processes in contrast to a spinning SFM that primarily causes reversals by a bottom-up, or stimulus driven, fast cycling through adaptation and recovery cycles. The proposed reason for this hypothesis is that wobbling SFM inhibit the adaptation-recovery cycles that are activated for spinning SFM. Efficient adaptation requires unchanging stimulus parameters, such as spinning direction and orientation of the cylinder. Reversals from observing the SFM stimuli are also compared with reversals from observing stationary and wobbling Necker cuboids believed to involve low level bottom-up driven processes [4–9]. One specific prediction that can be formulated is that if common processes underlie the reversals for these stimuli, then the reversal rates, or stable durations, should be correlated between stimuli. Scores from an imagery questionnaire that targets endogenous processes in the form of visual imagery vividness was also administered to estimate individual self-reports of visual imagery vividness, here used as a proxy for estimating the strength of top-down signals.

A number of studies provide support for the influence of bottom-up processes causing reversals while viewing ambiguous SFM. Some models propose that shifts in the balance between competing neural representations trigger perceptual reversals. Such shifts may be due to hard-wired processes such as adaptation [5, 6], neural noise [10], or changing the input by redirecting gaze [3, 11, 12], or bottom-up flow of prediction errors [13, 14]. That reversals from ambiguous spinning SFM-stimuli involves low-level hard-wired processes is suggested by priming [15–17], and adaptation studies [18, 19]. Change in activity in cortical area V5/MT coincide with reversals of the perceived spinning direction of cylindrical shapes defined by SFM, suggesting that this area play a crucial role in SFM perception [20]. Likewise, evidence exists for low-level processes involved in adaptation to line drawings such as the Schröder staircase [5, 6] and Necker cubes [4] (wire frame figures with ambiguous depth order). Local influences from adaptation is a hallmark of low level processes, and such local influences have been found in adaptation studies using Necker stimuli: Adaptation to a spinning Necker cube elevates reversals of a subsequently presented spinning cube, but only if presented at the same visual field and were of the same size as expected from low-level processes [21]. Also, adaptation to an disambiguated version of a static Necker cube causes a subsequent ambiguous cube to be seen in the opposite perspective, but the effect is local [22, 23].

Top-down processes also contribute to reversals as suggested already by Helmholtz [24] who claimed to have full control over the perceived interpretation of ambiguous stimuli by paying attention to either interpretation of it. Influencing ambiguous stimuli by will likely involve visual imagery which is known to influence perceived depth in SFM [3]. Visual mental imagery, perceiving things before the mind’s eye, plays an important role in in generative models, such as predictive processing where sensory information is analysed by comparing it to endogenously generated similar patterns [25]. Although reversals during prolonged viewing are hard to prevent suggesting hard wired processes [26, 27], reversal rates can be increased by knowledge of reversibility and instructions to switch between alternative perspectives as quickly as possible [28, 29]. Brain imaging also provides support for top-down influences by showing that perceptual shifts are accompanied by activity corresponding to shifts in top-down flow of signals (i.e. hypotheses) to explain the sensory information [30, 31].

Long et al. [32] proposed a two-stage model of reversals from observing spinning Necker cubes. This model is characterized by top-down influences from learning and bottom-up influences from adaptation-recovery cycles of two sets of neural populations that represent the two percepts of spinning direction. The duration of prior exposure to a disambiguated Necker cube influence whether priming or adaptation occur [4]: after brief exposure the subsequently presented ambiguous Necker cube is perceived in the same configuration (priming) and after long exposure it is perceived in the opposite configuration as the disambiguated Necker cube (adaptation). There is other evidence for experience-based influences in the perception of Necker cubes. The Necker-cube is often seen with the perspective in accordance with lying on a ground surface as seen from above rather than from seen from underneath due to a gravitational frame of reference [33], but after a long period of weightlessness astronauts perceive both interpretations for the same duration [34].

Different researchers investigating ambiguities have used different types of stimuli, but it is not evident that findings obtained using one type of stimulus can be generalised to other types of stimuli. The impacts of different processes could lead to the differences found regarding correlations between stable durations obtained from various ambiguous stimulus types [35]. Reversals caused by adaptation-recovery cycles between competing neural circuits should slow down if adaptation is hampered. Such cycles result from successively increased adaptation of the neural population supporting the perceived interpretation, while the neural population supporting the opposite interpretation recovers and finally takes over. Inhibitory couplings between these populations ensure that only one interpretation at a time is perceived. One way to prevent adaptation of low-level motion direction and orientation selective units is to oscillate the objects spinning direction and its orientation in space. This prevents long lasting non-varying spinning direction and object orientation, which is required for efficient adaptation. Accordingly, when frequently alternating the simulated rotation direction of a revolving SFM-shape, or when the simulated slant as signalled from velocity gradients in SFM oscillates back and forth with small amplitude during observation, then perceived reversal rates drop remarkably [2]. The impeded adaptation may then shift the main cause of reversals to other slower processes, such as switches between top-down hypotheses. Support for this comes from a recent study showing that brief presentation of a disambiguated stimulus (a prime) efficiently influence the perception of a successively presented ambiguous wobbling SFM. This priming is independent of location of the prime, and occur whether the prime is a disambiguated SFM, a static image, or specified by imagery alone [3]. It has been proposed that top-down processes in the form of visual imagery (perceiving before the mind’s eye) plays a role for the hypothesis generator that attempts to explain sensory information [36, 37]. Influences of imagery on perception have since then been reported using ambiguous figure-ground stimuli [38] and binocular rivalry [39]. Also, voluntary control of one type of reversible figure is highly correlated with the control over another type [26], but correlations between spontaneous switching rates obtained from different kinds of ambiguous stimuli are clustered demonstrating both shared and independent mechanisms [35]. Thus, there is evidence for contributions of a common top-down process, possibly related to imagery, in addition to stimulus specific low-level processes such as adaptation-recovery cycles.

The aim of this study is to probe bottom-up and top-down processes involved in the perception of ambiguous spinning and wobbling SFM stimuli. As a comparison two Necker type stimuli where used (wire frame cuboids), one was a still image and the other was wobbling with the same motion as the wobbling SFM. The spinning SFM stimulus was a cylinder with reversible spinning direction, hypothesized to target reversals primarily due to fast adaptation-recovery cycles between competing neural populations [4–8]. The other SFM stimulus consisted of a wobbling cylinder with an ambiguous sign of slant, hypothesised to prevent efficient adaptation and target slower top-down processes [3]. The still image was the classic static wire-frame drawing of an elongated Necker cuboid with bi-stable orientation [40]. The wobbling Necker cuboid was set in identical wobbling motion as the wobbling SFM cylinder, and is an example of the kinetic-depth effect initially presented as shadows of revolving wire frame figures or geometrical shapes [41]. The motion gives a very compelling impression of 3D structures. Reversals from the two Necker stimuli is believed to activate both low and high level processes in line with the two-process theory [4, 32]. A within subject design was used where participants reported occurrences of perceived reversals and mixed percepts during prolonged viewing of the ambiguous stimuli. Of special interest were possible differences in durations of stable percepts and correlations between durations obtained from the different stimuli. If the same underlying process is involved in producing reversals then correlations between stable percept durations is expected. Also investigated were possible biases and influences of repeated measurements. The vividness experienced during imagery, as scored by questionnaires, has been associated with higher activation of top-down connectivity [42, 43]. No correlation, however, was found in a previous study between priming, or reversals, of an ambiguous wobbling SFM stimulus, and imagery as scored by self-rating questionnaires [3]. This was surprising since the perception of the ambiguous stimulus could efficiently be influenced by instructing participants to imagine one specific interpretation of the stimulus before it appeared. An attempt to replicate and generalize this finding to other stimuli was made by administrating a visual imagery vividness questionnaire.

## Method

### Participants

Forty-five adults (mean age 28, std 14; 31 females) participated in this experiment and were compensated with a voucher. From a previous study it was determined that this sample size would be sufficient to detect the effect sizes sought for [2, 3]. The recruitment period and data collection started 2022-10-01 and was finished 2022-12-16.

### Ethical considerations

Participants gave written informed consent. Data was anonymized and no sensitive personal data was collected. The study involved no physical intervention on participants or biological material taken from the participants. The participants were neither physically nor psychologically manipulated, and there was no risk of harming the participants physically or psychologically. The participants were informed that they could terminate participation at any time during the experiment and still keep the voucher. According to the Swedish act concerning the Ethical Review of Research involving humans (2003:460), ethics approval is required if any of the above considerations are not fulfilled. Since these considerations were fulfilled, no ethics approval was required for this study. The study was conducted in accordance with the code of ethics of the World Medical Association (Declaration of Helsinki).

### Stimuli

**Fig 1:**
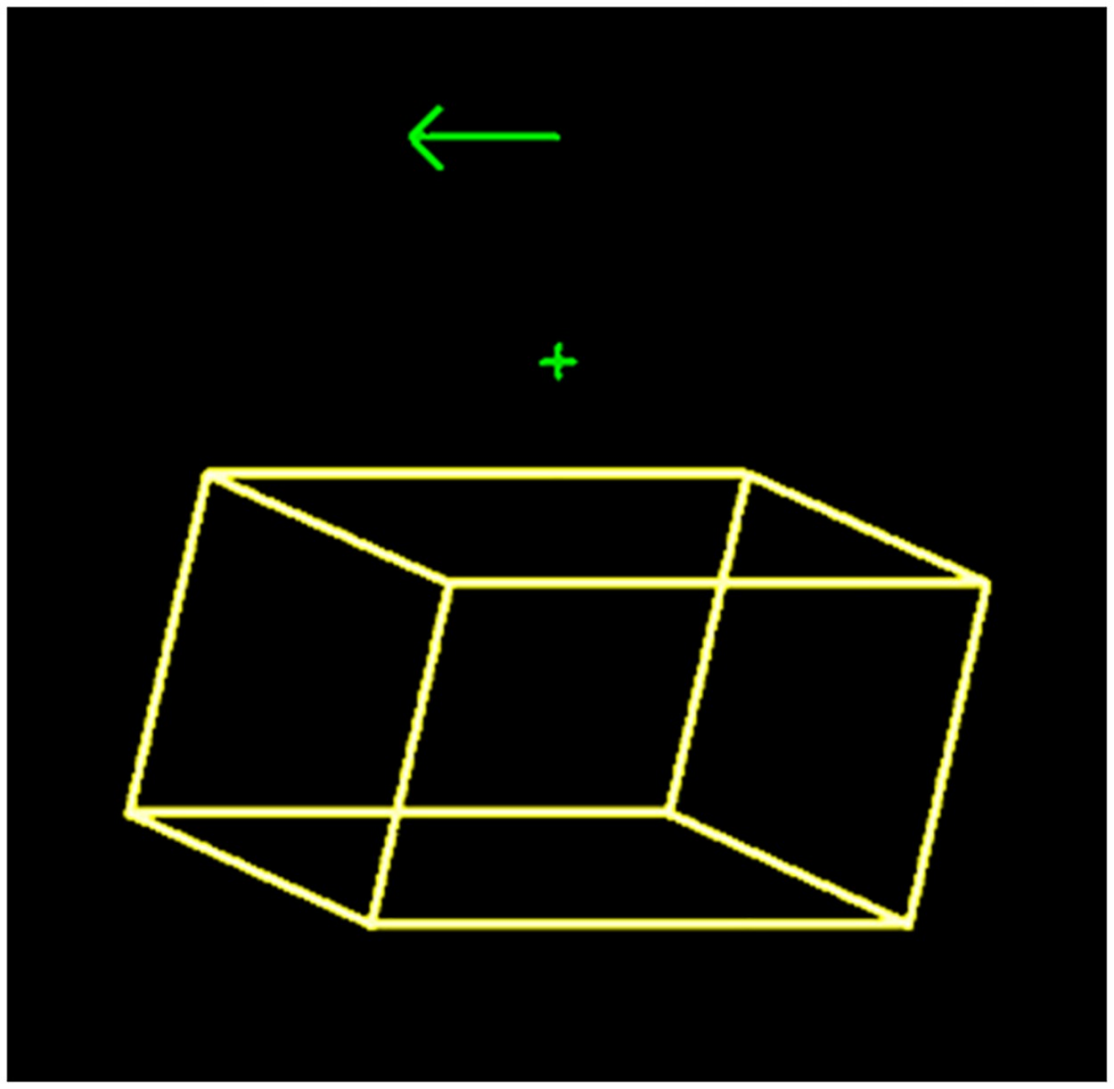
Stimulus example. The static Necker stimulus with the fixation cross and the arrow pointing leftwards to indicate the last response, as they appeared on the screen.

Stimuli were yellow (dots or lines) with RGB code (250, 250, 120) against a black (0, 0, 0) background. A green fixation cross was presented above each stimulus. Participants reported reversals between distinct percepts (left or right side closest, or spinning direction), and occurrences of mixed/unclear percepts. It was found in pre-experimental tests that participants were sometimes confused over which percept they had experienced just before an eye-blink. To support memory in these cases, the last response was indicated by a green arrow (pointing left or right), or a line without any arrowhead (for a mixed response). These appeared immediately above the fixation cross after each response (Fig 1).

**Fig 2:**
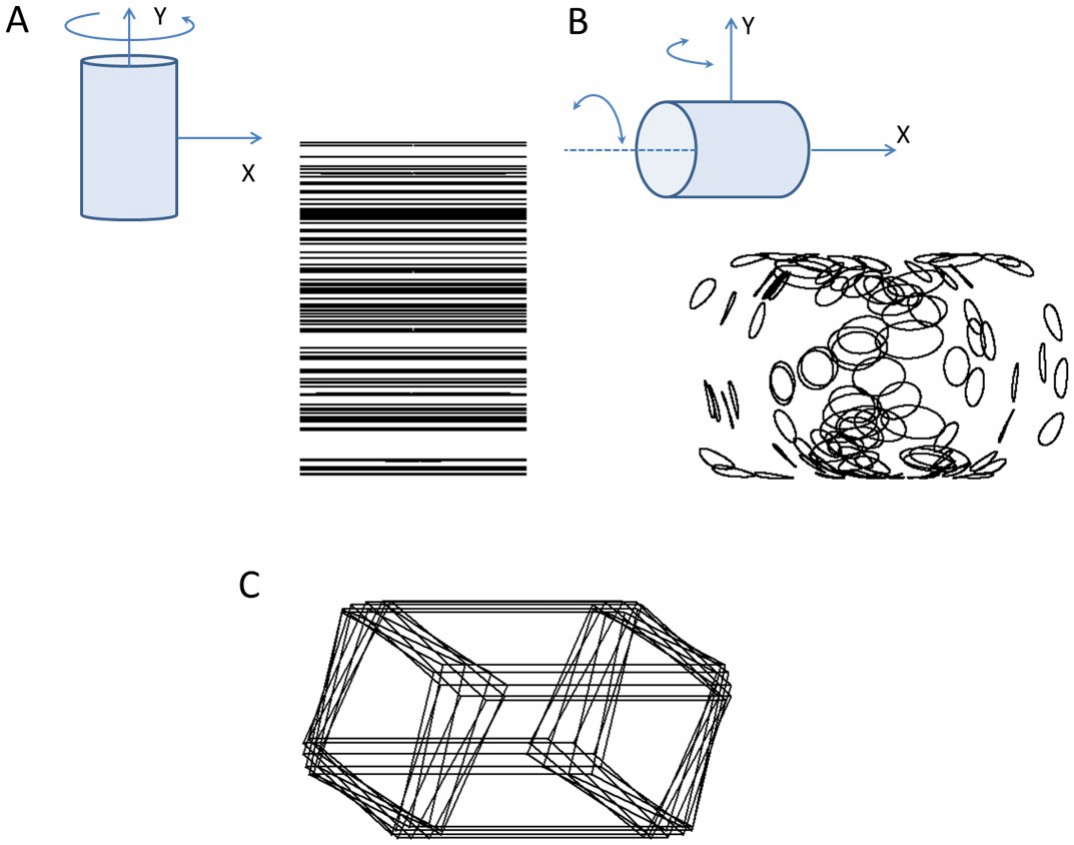
Schematics of moving stimuli. A. The schematic drawing on the upper left show the spinning SFM cylinder in vertical position revolving around its principal axis, and the resulting straight paths of parallel projections of the 100 dots moving back and forth as a result of the spinning, obtained with a long exposure. B. On the upper left is the wobbling SFM-cylinder oscillating around its vertical and principal axis, and the resulting elliptical shaped motion paths of the dots as displayed with a long exposure on the projection surface. C. The wobbling Necker-cuboid with 10 overlapping frames from the movie sequence (same motion as in B).

The SFM movie sequences were created by randomly spreading 200 dots randomly across the surface of a simulated cylindrical tube with radius 2.5 cm and length 7.5 cm (on the screen). The movie displaying the vertically oriented spinning SFM-cylinder was composed of 180 frames. These frames were created by sequentially rotating the dot coordinates around the main vertical axis with 2° steps between each frame. The rotation was accomplished by multiplying the dot coordinates with a rotation matrix. After each 2° rotation all dot coordinates were parallel projected onto the projection surface (coordinates on the screen) to be saved as a movie frame. In parallel projection the relative distance between dots on the surface of the SFM stimulus do not change as they move toward and away from the viewer (in contrast to perspective projection, where distances between dots would expand and shrink). This removes perspective cues that could otherwise disambiguate the depth relations. Algorithms for the rotation of coordinates in 3D and subsequent projection onto a 2D surface is provided by Poom [44]. All frames were then sequentially presented with 40 milliseconds stimulus interval so a complete revolution took 7.2 seconds (see demo spinning SFM, S1 Movie). Fig 2A shows a schematic illustration of the spinning cylinder with the frontal x and y-axes, and the motion paths of 100 dots obtained with a long exposure as they appeared on the screen, forming straight paths of oscillating motion along the horizontal.

The wobbling SFM-cylinder had the main axis aligned in the horizontal plane with a 45° tilt from the frontal plane. From this position, the dots were set into sinusoidal oscillation around the cylinder main axis synchronised with an oscillation around the vertical axis. This resulted in a wobbling motion of both direction and orientation of the cylinder that minimise adaptation to these features [2]. Both oscillation components had identical periods of two seconds, and equal amplitudes of 10°. One complete motion period was composed of 50 frames, sequentially presented with 40 milliseconds inter stimulus interval resulting in a duration of 2 seconds. The cylinder could be perceived with either it’s left or right opening directed towards the observer (see demo wobbling SFM, S2 Movie). Fig 2B shows a schematic illustration of the cylinder with its two components of oscillation and the frontal x- and y-axes, and the motion paths of 100 dots obtained with a long exposure, forming elliptical, or closed elongated motion paths. The speed of individual dots varied across a complete period for both the spinning and wobbling SFM cylinders. For the spinning cylinder dot speeds varied between a momentary speed of 0 cm/sec for dots at the rim of the cylinder, to 2.2 cm/sec for dots at the middle part of the cylinder. For the wobbling SFM cylinder the dot speed depended on the specific location of the dot and its phase in the motion sequence, and varied between 0 cm/sec (when switching direction at the rim of the cylinder) up to a speed of about 2.4 cm/sec (for dots in the middle part of the cylinder). Hence, the dot speeds varied within about the same interval for the two stimuli during the motion sequences.

The Necker figure was a wire frame drawing of a cuboid specified by the 3D coordinates of the eight vertexes (Fig 1). The wobbling cuboid was created by transforming the vertex coordinates in the same way as the wobbling SFM, and then joining the vertexes by lines (see demo wobbling Necker, S3 Movie). A complete motion period of 2 seconds was composed of 50 sequentially displayed frames with 40 milliseconds inter stimulus interval. Fig 2C shows 10 overlapping frames from the movie sequence (every fifth frame), to avoid cluttering.

### Procedure

The two SFM stimuli and the two Necker cuboid stimuli were presented sequentially in two sets run in a succession. The order between stimuli was randomized for each participant, and the same random order was used in both sets for each participant. This kept the time between the two presentations of the same stimulus to about 15 minutes across all four stimulus conditions, including reading brief written instructions presented on the screen before each new stimulus. Presentation times for each stimulus in both blocks were 150 sec, making a total of 5 minutes of observation per stimulus while reversals between distinct percepts and occurrences of mixed/unclear percepts were reported by key press. Before the experiment the participants were shown examples of the stimuli and the task was described. Participants were instructed to fixate the cross above the stimuli during the whole presentation time to reduce the possibility of stimulus-triggered or deliberate eye movements that could impact the switching behaviour [35].

Participants pressed the F-key when the rotation was perceived as clock-wise (the near side moving to the left), or the left side of the wobbling cylinder/ Necker cuboid was perceived closest. They pressed the K-key when anti clock-wise rotation direction was perceived, or the right side was perceived closest. The space bar was pressed when no clear direction or orientation could be seen (mixed percepts). Immediately after the F-key (for left), K-key (for right), or space-bar (for mixed percepts) was pressed, a left-arrow, a right-arrow, or a line without any arrowhead appeared above the fixation cross indicating the response. After the two sets of ambiguous stimuli were presented, the VOSI imagery-questionnaire was administered [45]. The VOSI questionnaire is a tool used to assess how well individuals can use endogenous processes devoid of sensory input to visualize images of objects and spatial relationships.

### Statistical analyses

Traditional frequentist p-values and Bayes factors (BFs) were calculated by a freely available statistical software [46]. The BF provides evidence both for and against the null-hypothesis and BF_10_ is defined as the ratio between the likelihoods of the results given H_1_ and H_0_ (BF_01_ is the inverse ratio). As a guideline it has been suggested that BF_10_ (BF_01_) between 1 and 3 (1-1/3) is considered as anecdotal evidence. A BF_10_ (BF_01_) from 3 to 10 (1/3–1/10) is considered moderate evidence, from 10 to 30 (1/10–1/30) as strong evidence, between 30 to 100 (1/30–1/100) as very strong, and beyond that extremely strong evidence [47]. All reported Bayes factors were computed using the default settings in JASP for the effect size priors. A flat prior for the alternative hypothesis stating that there is a true correlation, was used for the correlation analyses. The non-parametric Kendall Tau-b was used for the correlation analyses since this rank correlation coefficient is recommended for skewed data when samples are small, or when the data contain ties. All data set is provided as S1 Data.

## Results

**Fig 3:**
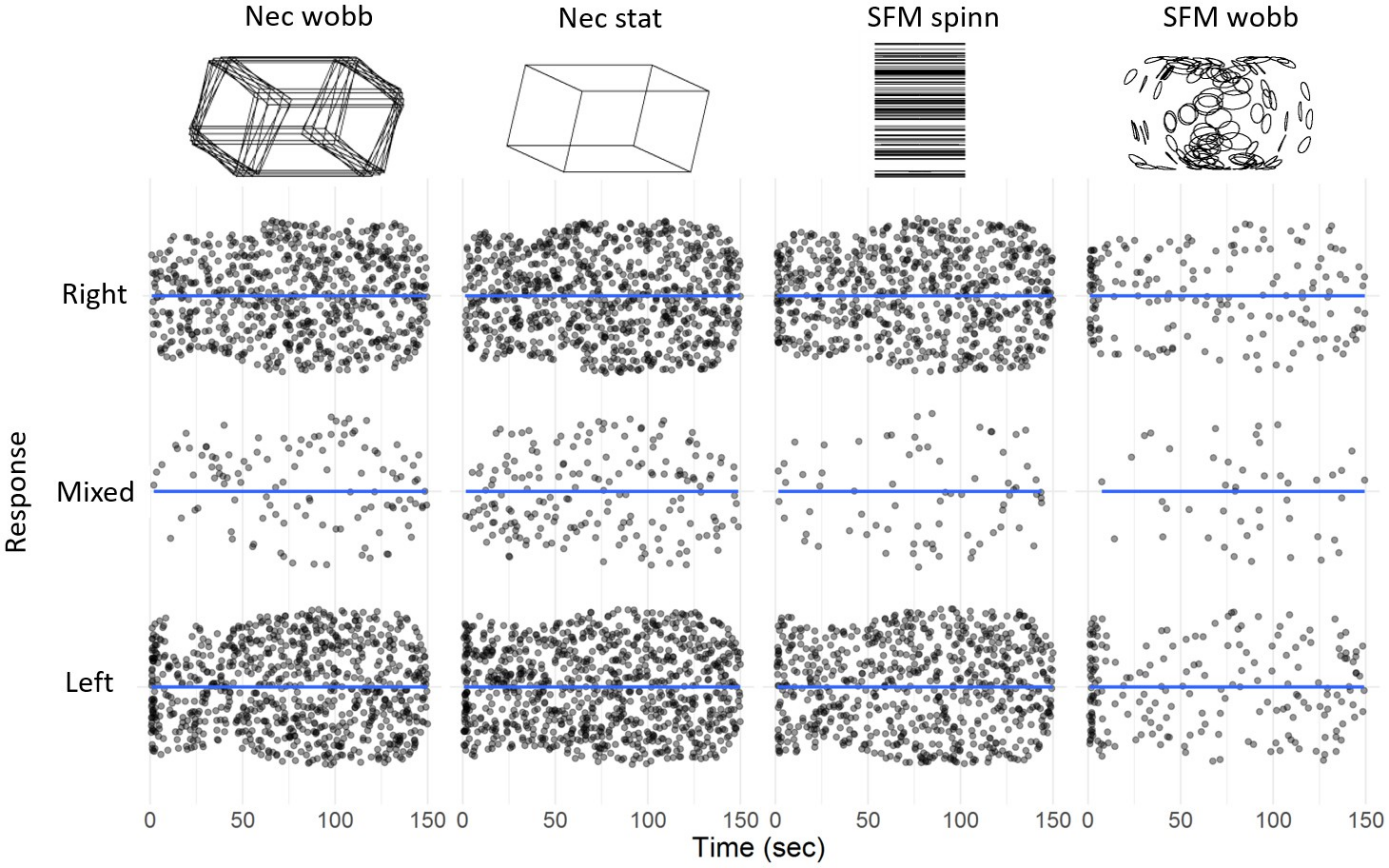
Flex-plots. Plots showing the time for change of percepts (*left*, *right* and *mixed* responses) after stimulus onset during the 2 x 150 sec observation for all observers. The four stimuli are presented in separate columns with data collapsed across the two sets in each flex-plot. The dots representing individual responses are spread along the dimensionless vertical axis to aid visualization of the density of responses at each time-unit after onset, darker regions indicate temporally overlapping responses.

Fig 3 present an overview of the data for the purpose of visual inspection. It shows the response patterns for all 45 participants over the two 150 sec sequences displayed as separate flex-plots for the different stimulus conditions (in separate columns). The number of responses is plotted across time and vertically spread to visualize patterns in the data. Apparent in this flex-plot are the overall fewer reversals observed from the wobbling SFM (the rightmost flex-plots) compared to the spinning SFM, and the two Necker stimuli. On average, across the two sets, for the wobbling SFM stimuli each observer reported only 2.0 reversals/minute, whereas 6.7 reversals/minute was reported for the spinning SFM, and 7.7 and 6.7 reversals/ minute was reported for the static and wobbling Necker stimuli respectively. The average number of mixed responses per minute was 0.32 for the wobbling SFM stimuli, 0.23 for the spinning SFM, and 1.6 and 1.0, for the static and wobbling Necker stimuli respectively. The first responses for both Necker stimuli seem to be biased toward reporting the left side of the ambiguous figure as closest. This is expected from the prediction that an influence of gravity influence perception, where the cuboids are seen as staying on a ground surface and seen from above rather than from underneath, as floating in the air.

**Fig 4:**
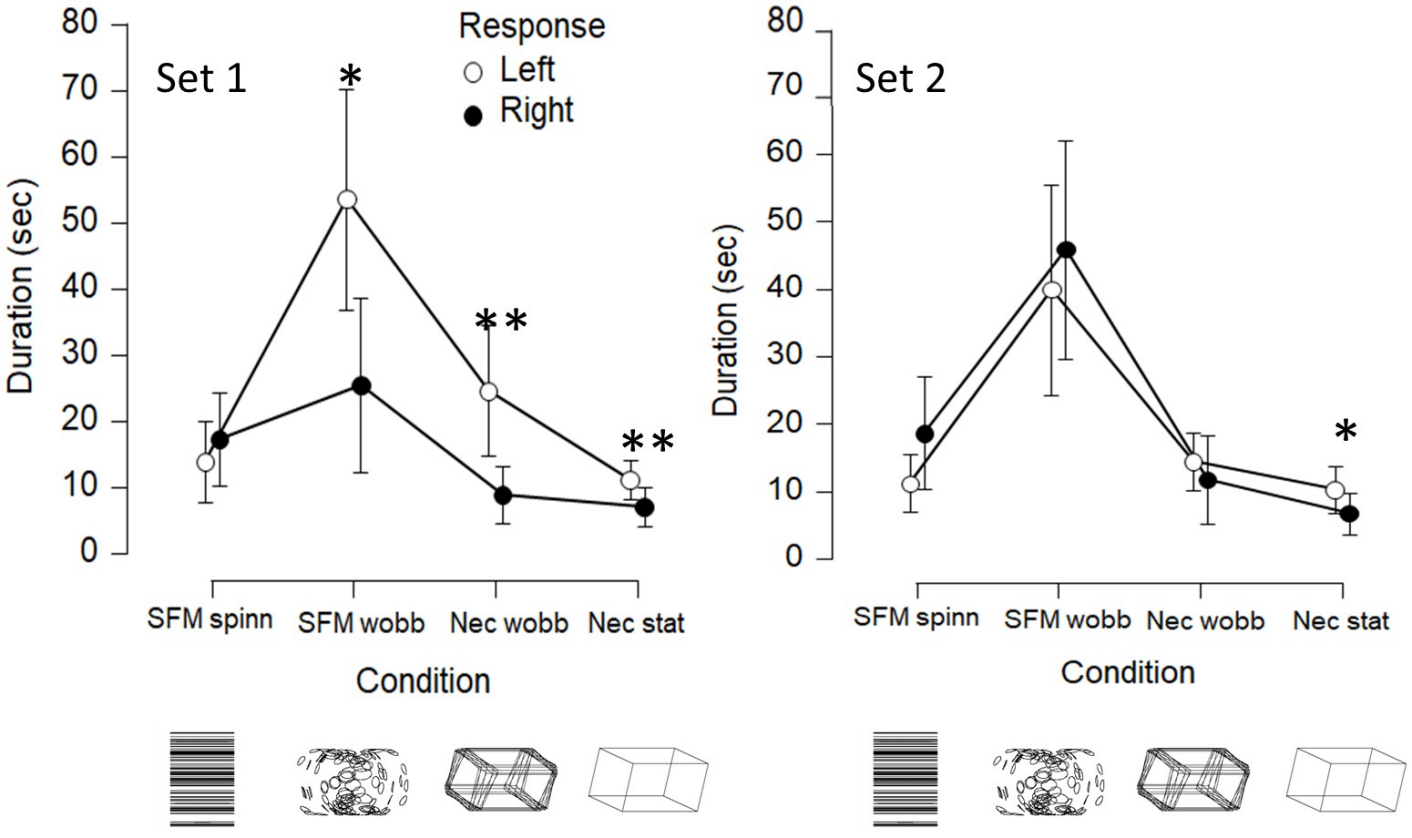
Mean durations. Shown are the durations of stable percepts in set 1 and 2. From left: the spinning structure-from-motion (SFM) cylinder with ambiguous direction (spinning in left or right direction), the wobbling SFM cylinder, the wobbling Necker cuboid, and the static cuboid with ambiguous orientation (left or right side nearest). Statistically significant difference between left and right durations, using a two-tailed test, are indicated by * (p < .05) and ** (p < .01). Error bars show the 95% CI.

Since mixed responses were few, the number of left and right responses are approximately equal within each condition, but the durations of left and right percepts may differ. In the following the duration of stable precepts was used as a dependent variable (results from analyses made using the number of responses led to the same conclusions as using the duration). Fig 4 shows the mean percept durations obtained in the four conditions in each set of 150 seconds observations (after averaging within participants first). Shapiro Wilks test suggested that distributions deviated from normality (durations were skewed). Therefore, the Wilcoxon two-tailed frequentist and Bayesian tests were used. The wobbling SFM stimulus result in much longer stable durations than for the other conditions, across comparisons with the wobbling SFM these differences were statistically significant, 959 < T < 1031, 4.98 < z < 5.80, all p’s < 0.001, 2044 < BF_10_ < 5.5 · 10^6^. The Bayes-factors indicate extremely strong evidence for longer stable durations obtained with the wobbling SFM compared to the other stimuli. The other three stimulus comparisons resulted in much smaller differences. Statistically significant differences in durations were obtained between the static Necker figure and spinning SFM (T = 742, z = 2.88, p = .004, BF_10_ = 13), and between the static Necker and wobbling Necker figure (T = 767, z = 2.81, p = .005, BF_10_ = 12). No statistically significant difference was obtained between the spinning SFM and the wobbling Necker figure (T = 552, z = .96, p = .34, BF_10_ = .28).

**Fig 5:**
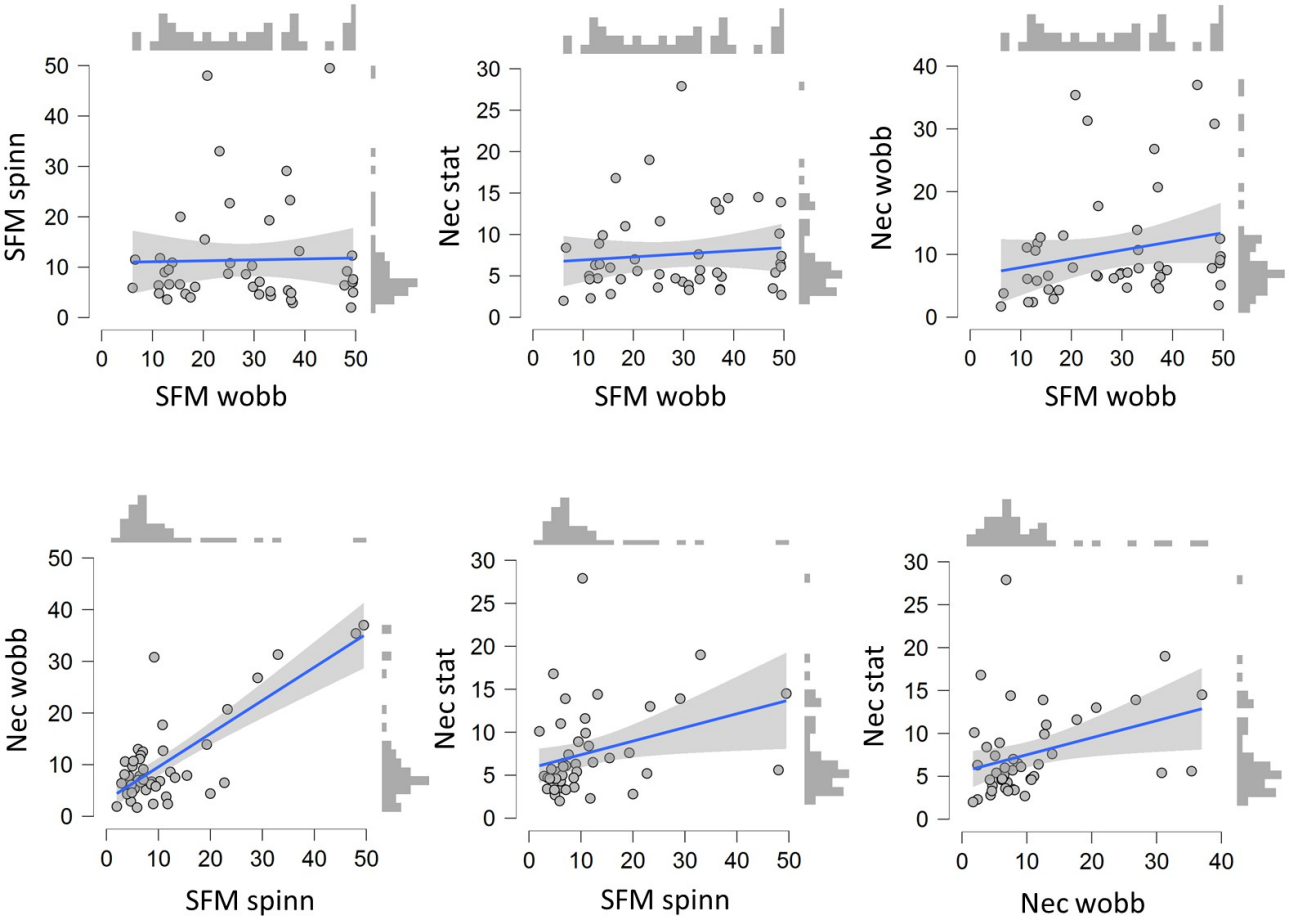
Scatterplots. Plotted are the mean (left, right, and mixed) durations in seconds for each of the 45 participants. Each pairwise combination of stimuli is shown in a separate panel (the wobbling and spinning SFM, and the wobbling and static Necker cuboid).

Fig 5 show scatterplots of stable durations for the pairwise stimulus conditions, with regression fits and duration-distributions. If the longer stable durations for the wobbling SFM is due to slowed down adaptation-recovery cycles, without involving any other slower process taking over, then a correlation between stable durations (or reversal rates) obtained from these stimuli are expected. The durations of stable percepts of the ambiguous SFM-wobbling was not correlated with neither the durations obtained from the SFM-spinning or the static Necker cuboid, and only weakly with the wobbling Necker cuboid (the three top panels in Fig 5). Table 1 show the Kendall’s Tau-b correlations between durations of stable percepts, and Bayes factors showing the ratios between the likelihoods of obtaining the result if there was a true positive correlation compared to if there was no correlation. No correlation was obtained between the spinning and wobbling SFM stimuli: Tau-b = -048, BF_10_ = 0.14 (this result is 7.1 times more likely given no true correlation than if there was a true positive correlation, using BF_01_ = 1/BF_10_). Weak evidence was obtained for a positive correlation between the wobbling SFM and the wobbling Necker conditions. In contrast, there is strong evidence for correlations between stable durations obtained from the spinning SFM and the two Necker conditions (BF_10_ > 20). In addition, visual inspection of Fig 5 shows that the duration of stable percepts in the ambiguous SFM orientation seem to be evenly distributed across individual mean durations; whereas the distributions of durations for the other three stimuli are highly skewed towards low durations. Thus, the idea that a common process mediates reversals obtained from the spinning (or from the other conditions) and wobbling SFM cannot be sustained.

**Table 1:** Bayesian Kendall’s Tau-b correlations.

		SFM spinn	Nec wobb	Nec stat
Nec wobb	Tau b	0.297 **		
	BF_10_	21.7		
Nec stat	Tau b	0.303 **	0.283 **	
	BF_10_	25.7	14.9	
SFM wobb	Tau b	-0.048	0.214 *	0.075
	BF_10_	0.14	3.08	0.38

Correlations between duration of stable percepts for all pairwise stimulus combinations, the spinning and wobbling SFM, and the static and wobbling Necker stimuli, for the 45 observers. All tests are one-tailed, for positive correlation

* p < .05,

** p < .01

The results from Poom and Matin [3] showing no correlation between a wobbling SFM and scores from imagery-vividness questionnaires were replicated and generalized to the spinning SFM and both Necker-stimuli. The durations of stable percepts and the VOSI scores for object or spatial imagery dimensions were not related. The correlations ranged between -0.15 < Tau-b < 0.11, and the Bayes factors ranged between 0.082 < BF_10_ < 0.55 (one-tailed test). This is interesting since imagery is an efficient prime for the wobbling SFM, where the ambiguous cylinder is perceived to be slanted similarly as an imagined slanted cylinder, as specified by instructions before the cylinder appeared [3]. This may suggest that imagery vividness as measured by questionnaires target other skills than real imagery online.

Subsidiary analyses were made to reveal possible biases of the interpretations of the stimuli. It was expected that a ground level bias, here resulting in a left duration bias, should exist for the Necker stimuli. Two-tailed Wilcoxon tests were performed within each condition in each set to test for any duration bias. Such statistically significant differences were found not only for the Necker cuboids, but unexpectedly also for the wobbling SFM (as marked by asterisk’s in Fig 4). The SFM stimulus contained no information that could be used to identify a ground level coupled with the left or right responses. Since the ground level bias and a left bias are congruent for the Necker cuboids it is not possible to distinguish these two biases from the duration data. The left duration bias decays in the second set presented 15 minutes later and occur in the first set for all slanted stimuli (The Necker stimuli and the wobbling SFM).

An additional subsidiary analysis showed that in the wobbling Necker condition, 84% of the first responses made across the two sets, across all participants, were “Left” responses. Correspondingly, in the static Necker condition 80% initial “Left” responses were obtained. For the wobbling SFM stimulus 59% of the initial responses indicate left side closest, and in the spinning SFM condition 50% indicated leftward rotation meaning that no bias was obtained. Thus, there was an initial bias for the Necker stimuli in that the left side was perceived as oriented toward the observer. Table 2 show a Bayesian Binomial test to see if the number of initial left responses differs from the 50% as expected if there was no bias, presented for the different conditions in sets 1 and 2. The probability of the results from both SFM-stimuli provide moderate evidence for no bias. In contrast, a large initial left bias was evident for the two Necker-stimuli. The BF’s are much greater in set 1 compared to set 2, but no statistically significant differences was obtained (p ≥ .22, the null model is specified as there being no difference between set 1 and set 2).

**Table 2:** Bayesian binomial test.

Condition	Set	Initial left responses	BF_10_	p-value
Nec wobb	1	38/45***	16 900	< .001
	2	36/45***	863	< .001
Nec stat	1	37/45***	3550	< .001
	2	34/45***	75	< .001
SFM spinn	1	22/45	.19	1
	2	22/45	.19	1
SFM wobb	1	26/45	.31	.37
	2	26/45	.31	.37

In separate columns are the number of initial left responses out of 45, the Bayes factors in support for a correlation, and p-values to find out if the numbers of initial left responses across all 45 participants differ from the expected proportion under the null hypothesis, i.e. 50%. All tests are two-tailed, i.e. the alternative hypothesis is that proportions ≠ 50%

**Fig 6:**
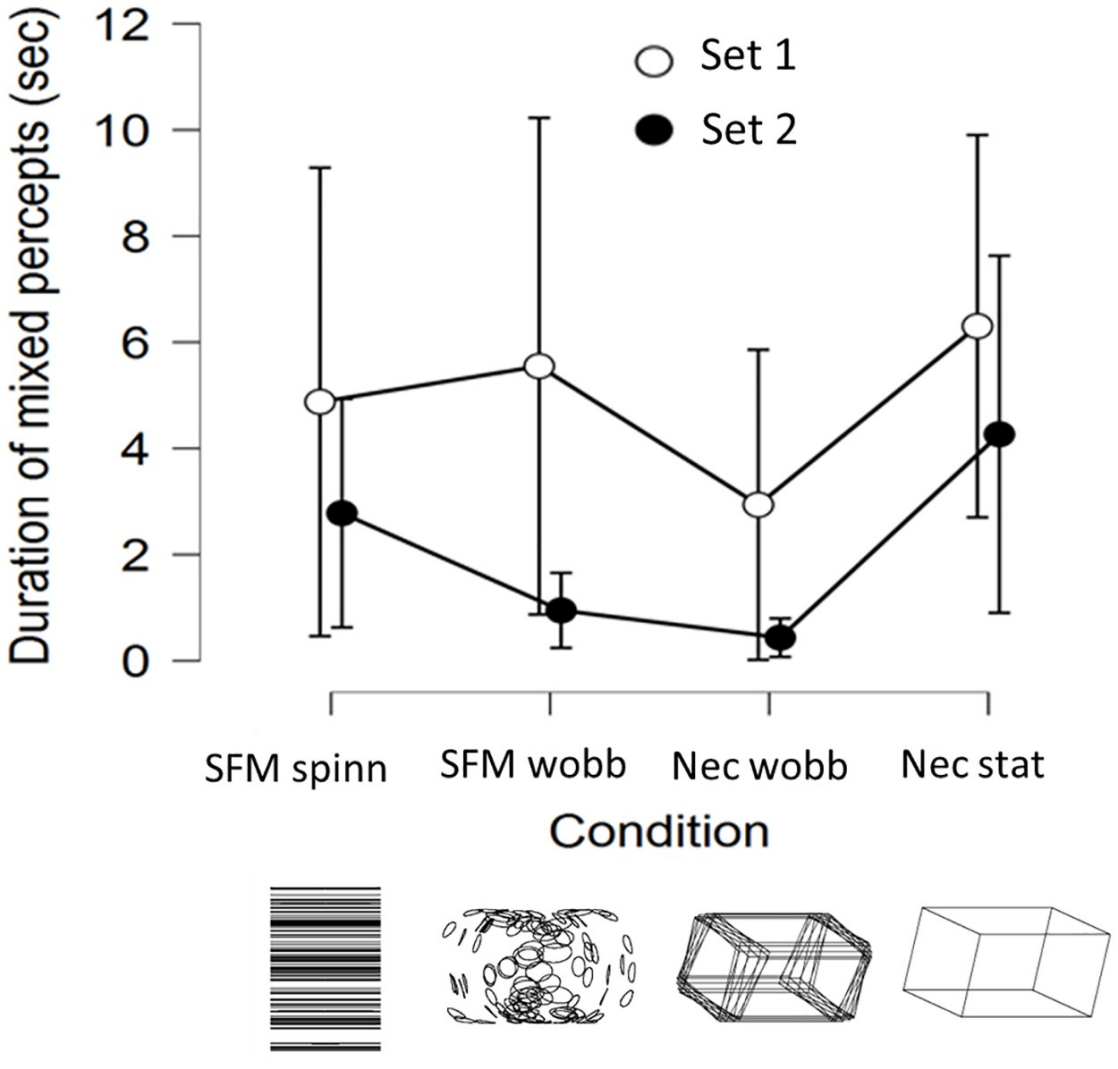
Mixed percepts. Mean duration of mixed/unclear percepts in set 1 and set 2 in the four stimulus conditions. Error bars show the 95% CI.

Over both sets and all conditions, mixed percepts were brief. Fig 6 shows that the mean duration of mixed percepts declined between set 1 and set 2. The Shapiro-Wilk test suggested a deviation from normality. Durations were skewed toward zero and the spread was different between sets, therefore the non-parametric Wilcoxon signed rank test was used. It showed that for mean durations across the four conditions there was a significant difference (Z = 3.61, p < 0.001) between durations of mixed percepts between the first and second sets. The evidence was classified as extremely strong by the Bayes factor (BF_10_ = 280). The mean and median duration of mixed responses in set 1 was 4.92 and 2.67 compared to 2.11 and 0.65 for set 2. When each condition was analysed separately only the durations of mixed percepts obtained with the SFM wobbling condition resulted in a significant difference between sets (Z = 2.45, p = 0.015.

## Discussion

The ambiguous wobbling SFM was perceived with longer stable durations between reversals than the spinning SFM and both the Necker stimuli. As found previously [3] the wobbling SFM reversed on average twice a minute, whereas the reversal rates for spinning SFM, the dynamic and the static Necker cuboid were 4 times faster (between 6.7 and 8.9 reversals per minute). The absence of correlations between the stable durations of the wobbling SFM and the spinning SFM, and the static Necker stimulus, support the hypothesis that the cause to the reversals from these stimuli are dominated by different processes. The wobbling motion of the Necker cuboid did not slow down reversal rates, possibly because the small amplitude may have failed to inhibit the adaptation-recovery cycle of ambiguous wire-frame figures. It is known that these stimuli activate broadly tuned orientation specific units with less precision than the corresponding SFM stimulus [5]. Stable durations of the wobbling SFM and the wobbling Necker stimulus were correlated, although supported by weak evidence from the Bayes factor. While a divergent slower process dominates the cause to the reversals for the wobbling SFM, a common process seems to mediate reversal obtained from the spinning SFM and both Necker figures. Stable durations from these three stimuli are similar and strongly correlated, individuals who experience fast reversals from the spinning SFM is also likely to experience fast reversals from the two Necker stimuli. In addition, for the wobbling SFM the distribution of mean stable percepts across participants are much wider and appeared uniform compared to mean durations obtained from the other stimuli that were skewed towards lower durations. Likely candidates for the processes acting as dominant causes to the reversals are low-level fast adaptation-recovery cycles, and a slower top down driven hypothesis generator that switches between the two possible explanations to the sensory stimulation.

Alternative causes to the different stable durations, such as dot speeds and the orientation difference between stimuli can be ruled out. It is unlikely that the orientation difference between the spinning and wobbling SFM cause the different reversal rates since in a previous study [2] similar high reversal rates was obtained with a spinning SFM oriented horizontally as the vertically oriented SFM used here. In the same study it was found that large variations in speed had little effect on reversal rates of SFM stimuli. Here the local dot speeds of the spinning and wobbling SFM were similar, so it is highly unlikely that that the large differences in reversal rates would result from the very small speed differences. The depth symmetry proposal [48], where the appearance of reversals of depth symmetric SFM-stimuli is a seamless transition, whereas the transition of depth asymmetric objects is experienced as a rough transition and therefore resist reversals, fail to account for the different reversal rates. Only the spinning SFM used here is perceptually depth symmetric and we would expect it to reverse more frequent than other stimuli used here. Specifically, the wobbling motion of the SFM and the dynamic Necker cuboid were identical and both give rise to a distinct asymmetric 3D percept, but reversal rates were similar to the depth symmetric spinning SFM.

Among subsidiary results was that an initial view-from-above bias was observed from observations of the Necker stimuli, which is well known from previous studies [33]. Similarly, a backward slant bias has been demonstrated from SFM-stimuli when the sign of slant, forward or backward, is ambiguous [2, 49]. The reason to this bias is consistent with our prior experience of gravity in which objects typically lie on the ground, below the eye level. Accordingly, after three months in weightlessness in orbit during space flight, the bias completely disappear and both interpretations are seen with equal probability [34]. No similar initial bias was observed from the other stimuli used here since their interpretations are not linked to any view-from-above, or view-from-below perspectives. An additional bias was observed, but only in the first set for the stimuli with the wobbling SFM, and both Necker cuboids, all with ambiguous orientation. In the first set these stimuli were perceived with a left duration bias, so the left side was perceived closer to the observer under longer durations than the right side was perceived as closer. This left duration bias could be a consequence of the well-known attention to the left bias [50]. Although observers were instructed to fixate the cross above each stimulus, they may have failed to resist directing overt or covert attention to the left side. The fixated part of the ambiguous Necker cubes [11, 12], and slanted SFM cylinders [3] therefore tend to be perceived as closest to the viewer. Contrary to the initial gravitational bias observed for the Necker stimuli the duration bias declined in the second set. Although the overall occurrences of mixed, or unclear, percepts were rare in line with previous reports [3, 51], the durations of mixed percepts also declined between sets. Durations were 4–6 seconds in the first set and 0–4 seconds in the second set (Fig 5). The differences between the two successively presented sets are clear demonstrations of fast influences of prior exposures on perception of these ambiguous stimuli.

Reversals may involve both stimulus driven bottom-up low-level processes, and endogenously generated top-down processes. Different types of stimuli could activate different processes to various degrees. Particularly, perception of spinning SFM involves low level processes where competing direction selective neural populations cycling through adaptation and recovery [52, 53] as suggested by priming [15–17] and adaptation studies [18, 19]. Similar results are found with Necker stimuli [7–9]. Such low-level explanation involving adaptation requires prolonged non-varying simulation which is prevented if the sign of direction, or slant, oscillates back and forth. If the wobbling SFM only led to a general slower adaptation-recovery cycle, with no other process becoming dominant, we would expect the durations to correlate with the durations from the spinning SFM. In addition, priming of the wobbling SFM, in contrast to the spinning SFM, is independent of the information used to disambiguate the prime, and the position of the prime which are hallmarks of higher-level processes [3]. Thus, there is evidence for that other slower top-down processes are targeted by preventing efficient adaptation of an ambiguous SFM stimulus. Future brain imaging studies using spinning and wobbling SFM stimuli could provide information about the neural correlates to the perceived reversals during observation of these SFM-stimuli.

It has been proposed that visual imagery plays a role for the hypothesis generator that attempts to explain sensory information [37, 54]. Thus, it might be suspected that if reversals are the result of such hypothesis testing then imagery as scored by questionnaires and reversals would correlate, but no such correlation was found. This is in line with previous work where no correlation was obtained between self-rated vividness of visual imagery, and the influence from an imagined prime, or the frequency of reversals [3]. Although imagery-vividness has been associated with higher activation of top-down connectivity [42, 43], it is not just a weak form of perception. The distribution of object information across visual areas is strikingly different during imagery and perception [55], and self-rated visual imagery as scored by questionnaires may not be strongly related to perceptual processes.

**Fig 7:**
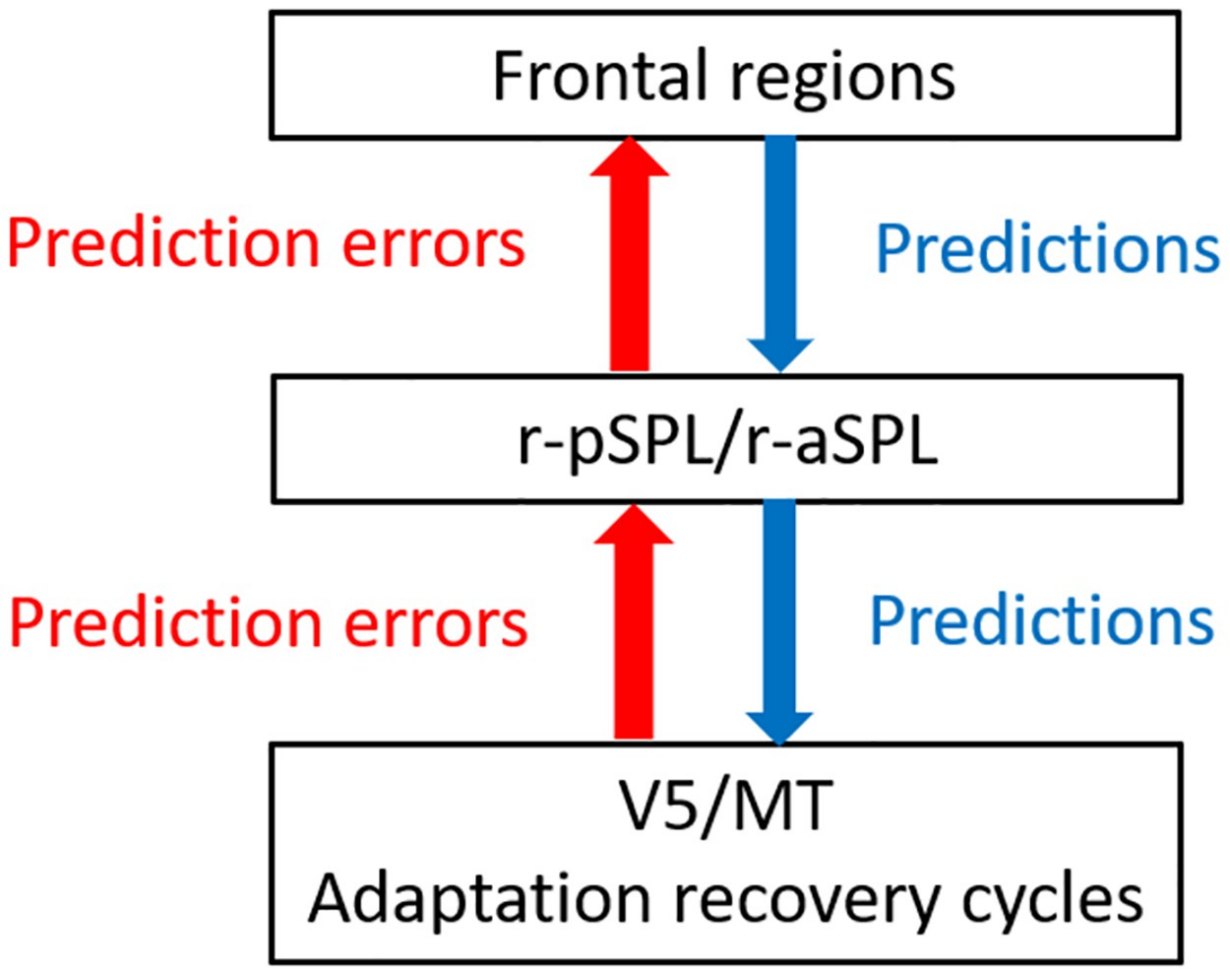
A conceptual model. A simplified predictive processing model describing the processes of perception and reversals from observing ambiguous SFM (see text for details).

The inferential predictive processing framework describes perception as a balancing act between bottom-up flow of prediction errors and top-down flow of hypotheses generated in an attempt to explain sensory data [13, 14]. Both processes may contribute to reversals [56, 57]. Accordingly, ambiguous Necker stimuli elevates both top-down and bottom-up activity compared to stable versions of these figures [31], and dynamic causal modelling provide support similar contributions to perceived reversals from a spinning SFM [13]. The causal modelling involve posterior and anterior superior parietal cortex (SPL) [58] and may also involve frontal areas [14]. As shown in Fig 7, the bottom level of this hierarchical network is the motion sensitive visual area V5/MT, comprised of neurones that signals a particular direction of motion at a particular depth-plane in response to either binocular disparity or SFM stimulation [59]. This area is likely involved in disparity-priming [17] and disparity-adaptation of ambiguously spinning SFM [18, 19]. In addition, the neural activity in this area coincides with reversals of the perceived SFM spinning direction [20]. There is also evidence that neurones in V5/MT are involved in detecting motion gradients [60, 61] and disparity gradients [61] for estimating surface slant. This suggests that area MT has a basic role in SFM perception, and that reversals for spinning SFM, due to adaptation-recovery cycles, seem likely to originate in area V5/MT [17]. When the adaptation-recovery cycles are impaired, then the slower process of top-down and bottom-up flow of predictions and prediction errors will have a stronger influence on reversals.

That different processes are involved for different kinds of ambiguous stimuli and even wobbling and spinning SFM is theoretically interesting, but it is also important to consider when using measures of reversals as a probe to endogenous processes, or when investigating relations of such perceptual processes to personality traits and clinical diagnoses. Researchers should consider if the hypothesised common neural process underlying personality traits and perception involve high- or low-level prosses. Previous research have demonstrated relations between intelligence and switching rate from binocular rivalry [62], the Big-5 trait openness and occurrences of mixed percepts in binocular rivalry [63], but not reversals [64]. Similarly, a positive correlation was found between openness and mixed percepts using the SFM-wobbling stimulus [3]. Lower reversal rates in binocular rivalry seem to be associated with higher levels of self-discipline [65], and introvert scores as reported in studies using figure-ground ambiguity [66] and reversible 2D rotation [67], whereas higher reversal rates for introverts is reported using a three-dimensional reversible figure [67]. Clinically diagnosed autistic people experience lower switching rates during rivalry [68] and lower reversal rates when observing Necker cubes [69] and ambiguously revolving SFM-stimuli [51]. It is unclear whether these relations are due to common bottom-up or top-down driven processes. Relations between switching rates and personality traits could result from variations in neurotransmitter activity causing variations in the rate of adaptation to visual stimuli. For example, anxiousness, a marker for the Big-5 trait neuroticism has been linked to higher switching rates when viewing binocular rivalry stimuli [70], and a selective agonist to a specific serotonin receptor slowed down switching rates [71].

To sum up, this study explored the diverse processes involved in triggering perceived reversals while viewing ambiguous SFM stimuli characterized by spinning and wobbling motion, and static and wobbling Necker stimuli in comparison. Previous research on reversals from SFM have typically used spinning SFM which involve low-level adaptation-recovery alternations between neural populations. The wobbling SFM stimuli elicited significantly longer stable durations between reversals compared to the other stimuli. Notably, the durations for wobbling SFM stimuli did not correlate with spinning SFM or the two Necker stimuli, suggesting distinct underlying processes. In contrast, correlations were identified between spinning SFM and the two Necker stimuli. The prolonged stable durations observed with wobbling SFM and the lack of correlations with durations obtained by the spinning SFM suggest efficient deactivation of bottom-up driven adaptation and recovery cycles, emphasizing the involvement of alternative contributions, such as slower top-down processes. Furthermore, the disappearance of biases observed in the second set implies influences of learning between sets, adding a dynamic dimension to the study. This research provides valuable insights into the complex interplay between bottom-up driven adaptation-recovery cycles and top-down processes in ambiguous perception. By elucidating the distinct contributions of different processes involved in organizing visual input and interpreting ambiguities, this study contributes to the broader understanding of the mechanisms governing visual perception and interpreting ambiguities.

## Supporting information

S1 Movie Demo of the ambiguous spinning SFM stimulus. (MP4)

S2 Movie Demo of the ambiguous wobbling SFM stimulus. (MP4)

S3 Movie Demo of the ambiguous wobbling Necker stimulus. (MP4)

S1 Data Sheet. (XLSX)
